# Imaging Genetics Towards a Refined Diagnosis of Schizophrenia

**DOI:** 10.3389/fpsyt.2019.00494

**Published:** 2019-07-12

**Authors:** Wenhao Jiang, Tricia Z. King, Jessica A. Turner

**Affiliations:** ^1^Department of Psychology and the Neuroscience Institute, Georgia State University, Atlanta, GA, United States; ^2^Mind Research Network, Albuquerque, NM, United States

**Keywords:** imaging genetics, diagnostic catalogues, heterogeneity, genetic overlap, brain alterations

## Abstract

Current diagnoses of schizophrenia and related psychiatric disorders are classified by phenomenological principles and clinical descriptions while ruling out other symptoms and conditions. Specific biomarkers are needed to assist the current diagnostic system. However, complicated gene and environment interactions induce great disease heterogeneity. This unclear etiology and heterogeneity raise difficulties in distinguishing schizophrenia-related effects. Simultaneously, the overlap in symptoms, genetic variations, and brain alterations in schizophrenia and related psychiatric disorders raises similar difficulties in determining disease-specific effects. Imaging genetics is a unique methodology to assess the impact of genetic factors on both brain structure and function. More importantly, imaging genetics builds a bridge to understand the behavioral and clinical implications of genetics and neuroimaging. By characterizing and quantifying the brain measures affected in psychiatric disorders, imaging genetics is contributing to identifying potential biomarkers for schizophrenia and related disorders. To date, candidate gene analysis, genome-wide association studies, polygenetic risk score analysis, and large-scale collaborative studies have made contributions to the understanding of schizophrenia with the potential to serve as biomarkers. Despite limitations, imaging genetics remains promising as more aggregative, clustering methods and imaging genetics-compatible clinical assessments are employed in future studies. We review imaging genetics’ contribution to our understanding of the heterogeneity within schizophrenia and the commonalities across schizophrenia and other diagnostic borders, and we will discuss whether imaging genetics is ready to form its own diagnostic system.

## Introduction

The current diagnosis of schizophrenia and psychiatric disorders is mainly based on phenomenological observation and clinical descriptions. Although these descriptions are reliable, they are not established on valid pathological bases (1). The heterogeneity of the symptoms, treatment response, and outcomes implies that there are different subtypes within schizophrenia, while phenomenological observation fails to generate precise subgroups revealing etiological and pathological differences (2). Additionally, similar psychotic symptoms aggregate in different disorders and in families. Behind this aggregation, shared biological mechanisms including genetics and neurophysiology are found (1). These findings suggest that the boundaries of psychiatric disorders are merging beyond the traditional categorical diagnostic system. The precise subgroups and disorder boundaries may optimize treatment and prognosis, and research-based biomarkers may help to fulfill this goal. Importantly, efforts have already been made as part of the *Diagnostic and Statistical Manual of Mental Disorders, Fifth Edition* (DSM-5).

Combining genetics and imaging to assess accumulating genetic variations on brain function and morphometry has become the integrated research method known as imaging genetics (3). Imaging genetics not only serves as a tool to understand the impact of genetic variations on both structural and functional brain, but it also enables researchers to capture the behavioral implication of those genes and associated brain alterations (4). Importantly, imaging genetics characterizes different pathways from genes, imaging, and behavior data. Its quantified findings make it possible to contribute to the currently unknown map of future diagnosis (5). The common technologies in imaging genetic include candidate gene analysis, genome-wide association study (GWAS) using imaging phenotypes, polygenic approaches (polygenic scores, pathway analysis, and multivariate methods), and developing novel approaches (6–8). We focus in this paper on the subset of imaging genetics that focuses on the relationships from gene to brain to behavior, which have generally focused on common variants in the single nucleotide polymorphisms (SNPs).

**Figure 1:**
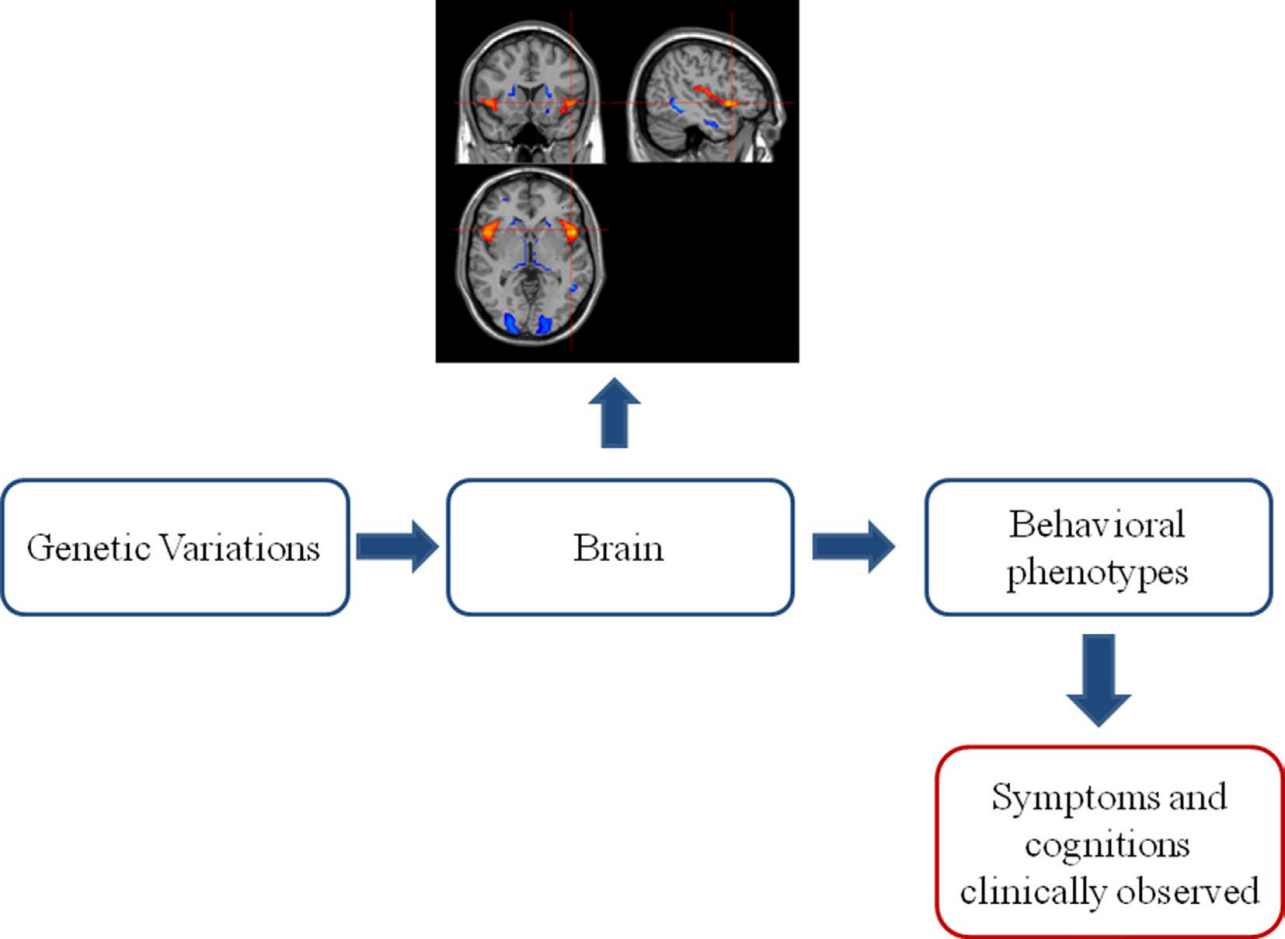
The classic “bottom–up” model in imaging genetics. Genetic variations acclimate their influences on the brain. The brain alterations further develop into behavioral phenotype changes, which can be clinically observed as symptoms and cognitive impairments. This observed clinical profile established the base of current phenomenological diagnostic system of psychiatric disorders.

Various genetically related brain abnormalities have been revealed in SZ. SZ patients generally show smaller brain volume, overall reductions in gray matter in fronto-temporal, thalamo-cortical, and subcortical-limbic circuits and enlargement of ventricles (9). These brain alterations induced in partly by genetic variations (10) are bridging the gap between gene and the phenotype and even clinical symptoms of SZ (see **Figure 1**) (11). It is encouraging that some shared genetics, imaging, and imaging genetics findings have been recognized across SZ, bipolar disorder (BD), and disorders under other categories. At the same time, imaging and genetics are helping to form subtypes with different mechanisms in SZ.

In this paper, we review the major imaging genetics findings on SZ with closely related psychotic disorders with an eye toward the following questions: 1) to date, what contribution have genetics, neuroimaging, and imaging genetics made to our understanding of the heterogeneity of SZ and the boundaries among psychiatric disorders and 2) whether imaging genetics is ready to form its own diagnostic system.

## Traditional and Current Diagnoses of SZ

Feighner and colleagues published the criteria for highly reproducible diagnoses based on behavioral observation in 1972 (12). From this historical view, clinical description, laboratory studies, delineation from other disorders, follow-up studies with retreatment response, and family studies are considered as major theoretical bases for validating a diagnosis (13). However, the follow-up study and treatment response may be questioned for whether they could validate the diagnosis, *per se*. Antipsychotics are a major choice not only for SZ spectrum but also for depression and BD.

Accumulating new biological understanding does not always agree with the criteria and classification proposed at Feighner’s time. It is now accepted that family coaggregation implies shared abnormal genetic markers and mechanism in the family line. For example, SZ, BD, and schizoaffective disorder (SAD) are in different diagnostic categories, but observations of diagnoses in families of patients showed significant overlap among them, which is still being studied to explore the genetic background (14). Thus, the traditional methods for determining the diagnostic category boundaries are not sufficient.

## Genetics and its Impact on the Diagnostic Problem

### Genetic Overlap Among SZ and Other Psychiatric Disorders

Genes contribute greatly to the etiology of SZ, and meta-analysis in SZ twin study shows a heritability around 80% (15). Val158Met single polymorphism (SNP) of catechol-O-methyltransferase (COMT), the Val66Met SNP of brain-derived neurotrophic factor (BDNF), and the Ser704Cys SNP of disrupted-in-SZ 1 (DISC1) is the most well-known gene alteration examined by candidate gene analysis (7). The first few reports of GWAS, in contrast, demonstrated several loci associated with SZ including Zinc finger protein 804A (ZNF804A), neurogranin (NRGN), and the major histocompatibility complex (MHC) region. More recent GWAS studies with increased sample size discovered more SZ related loci (16), and some of these loci are shared by BD and other psychiatric disorders (17–25).

SZ and BD are often studied together to elucidate the genetic overlap and disorder boundaries. A genetic correlation around 0.6 is suggested by family, twin, and adoption study (26). However, applying a hierarchical or nonhierarchical diagnostic system has provided conflicting co-occurrence results at the same time (27). SAD is often included in the study of SZ and BD and that genetic relationship could also be potentially affected (28).

In addition to epidemiological evidence, the GWAS study has brought more insight into the actual genetic overlap. ZNF804A is the first discovered marker that may increase the risk for both SZ and BD, and meta-analysis has supported its role (29). The combined SZ and BD GWAS study from Psychiatric Genomics Consortium (PGC) has identified calcium voltage-gated channel subunit alpha1 C (CACNA1C), ankyrin-3(ANK3) and inter-alpha-trypsin inhibitor heavy chain 3–4 (ITIH3-ITIH4) as risk for both disorders (30, 31). Later by introducing pleiotropy-informed conditional false discovery rate, 14 loci were associated with both disorders, and CACNA1C and ITIH4 were identified again (32). PGC’s diagnostic specificity of five disorders analyses has also shown 5′-nucleotidase, cytosolic II (NT5C2), and coiled-coil domain containing 68 (CCDC68) is associated with both disorders (33). The combined GWAS studies will continue to reveal more important loci, but the functional implications and roles of these distinct genes in SZ and BD will need further investigation.

Another idea is using a polygenetic method to combine and count the accumulating effects of a large number of loci, which may or may not reach the GWAS threshold for significance. Again in the PGC study, the cross-disorder group stated SZ and BD were affected by genetic correlation of 0.68 based on their common SNPs (33). Additional polygenic studies blur the distinction across categories and indicate a broad genetic mechanism for these psychiatric disorders (34–36).

However, there is also genetic evidence showing distinctions between SZ and BD (37). Large and rare copy number variations (CNV) have been identified in SZ and certain developmental disorders, but less consistently in BD. In addition, Sz pathogenic CNV carriers showed reduced subcortical regions including thalamus, putamen, pallidum, hippocampus, and accumbens, which were previously identified in Sz participants (38). This finding is consistent with the diagnosis hierarchy, by which BD is only diagnosed with the absence of SZ and developmental disorders.

### Genetics Helps Reveal Heterogeneity and Future Subtypes of SZ

Many researchers have tried to provide genetic explanations for SZ’s heterogeneities. Arnedo and colleagues made a promising attempt trying to uncover the hidden genetic architecture of different subtypes of SZ (39). The basic idea of their research was to measure the complexity of hidden architecture in genotype and phenotype. It was expected that the association between distinct sets of phenotypes and SNPs could be revealed in heterogeneous SZ, and it would represent subtypes of SZ with the respective genetic mechanism.

Arnedo et al. generated phenotypic sets using non-negative matrix factorization from the data of series questionnaire and structured interview results. The factorization divided the SZ patients into distinct subgroups with different disease severity, process, and symptom domain (positive, negative, and disorganized symptoms) regardless of their genetic background. SNP sets were generated by a generalized factorization method combined with non-negative matrix factorization. The overlap of patients and SNPs in these sets ensured to be disjoint, to reflect the heterogeneity of SZ. Finally, the association between phenotypic sets and SNP sets were tested in the molecular genetics of schizophrenia (MGS) study. It was also largely replicated by them in the National Institute of Mental Health Clinical Antipsychotic Trials of Intervention Effectiveness (CATIE) project and Portuguese Island family samples.

The results were encouraging: Arnedo et al. found 42 SNP sets had >70% risk for SZ, and these SNP sets were significantly associated with different phenotypic sets. For instance, a phenotypic set indicating a general process of severe deterioration (severe process, with positive and negative symptom; moderate severity of impairment; unable to function since onset) was highly correlated with certain SNP set including polypyrimidine tract binding protein 2 (PTBP2) and several other genes which might play a role in neuron differentiation. This severe deterioration SZ may be a potential clinical valid subtype, and following the track of PTBP2 and its SNP cluster may facilitate the examination of the mechanisms underlying severe deterioration.

Based on their findings, it was believed that SZ could be seen as “syndromes group” in which distinct clinical syndromes are associated with disjoint genotypic networks. The interaction map of disjoint genotype and distinct syndromes have shown a possible way of shaping SZ into biological markers or a networks-based subtype.

## Imaging and Its Impact on the Diagnostic Issue

Anatomical changes in fronto-temporal, thalamo-cortical, subcortical-limbic circuits, enlargement of ventricles, and widespread white matter fibers abnormalities have been found in many structural studies of schizophrenia (40–43). With the growing sample size and collaboration through different sites, many large-scale meta-analyses have provided new information. The Enhancing Neuroimaging Genetics through Meta-Analysis (ENIGMA) SZ working group’s meta-analysis of subcortical regions across several thousand subjects reported the consistent findings of smaller hippocampus, amygdala, thalamus, accumbens, and intracranial volumes, but larger pallidum and lateral ventricle volumes (44). The putamen and caudate volume results were not reliable across different populations and studies even with this sample size, indicating the possibility of clinical heterogeneity affecting those regions (44). The development of these differences prior to, with, and after disease onset and diagnosis is also important for understanding the disease (45), and comparing the course of the morphometric reductions and increases across diagnoses will be informative. Functional imaging studies have also discovered various abnormal brain regions and connections in SZ. Partially overlapped with structural findings, functional alterations including the prefrontal cortex, superior temporal gyrus, thalamus, frontal lobe, and parietal lobe have been reported in either resting state or task fMRI (46).

Many of the above regions have been identified as structural or functional commonalities among DSM categories (1, 47, 48). Starting from the same point as genetics, there are also imaging research efforts trying to redraw the boundaries between psychotic disorders. One pioneer study is from the Bipolar-SZ Network on Intermediate Phenotypes (B-SNIP) Consortium, Clementz et al. applied neurobiological measures among SZ, BD, and SAD and tried to regroup them into different “biotypes” rather than DSM catalogs (49). A selection of psychotic biomarkers and functional brain activity were collected in this study. Not only patients with psychosis but also their first-degree relatives and healthy controls were included. Clementz et al. then identified three “biotypes,” which were also believed to be more heritable than their original DSM diagnoses. Sensorimotor reactivity and cognitive control distinguish three biotypes: biotype 1 patients showed serious impairment across sensorimotor reactivity and cognitive control; biotype 2 patients show only deficits in cognitive control; and biotype 3 patients seem to be the mildest in cognitive symptoms. The B-SNIP group has also been trying to find the factors that contribute to its biotyping; one attempt is using the flow–frequency fluctuations (ALFF/fALFF) across the SZ, BD, and SAD from the large B-SNIP family study (50). More recently, gray matter density was checked in these three biotypes, and the density loss followed the same order as cognitive decline: biotype 1 showed whole brain gray matter density loss, while type 2 showed largely overlapping results with type 1, and the largest effects were found in fronto-temporal circuits, parietal cortex, and cerebellum. The findings were much more localized and of less magnitude for type 1. Type 3 only showed small reductions in frontal, cingulate, and temporal regions despite their similar DSM diagnoses (51).

## Imaging Genetics to Refine the Diagnosis

### Imaging Genetics Linking Genetics, Intermediate Imaging, and Cognitive Phenotypes

There are hundreds of papers using imaging genetics method to study SZ in the past 10 years, but here, we will focus on the findings with relatively clear functional implications. First, we selected the genes that have been highlighted in SZ and cognitive functions and if there is more than one report implicating those genes. The details of included genes can be found in **Tables 1** and **2** under “Risk SNPs/allele” column. We then searched PubMed database using the terms: [“gene symbols”] (genes we selected) AND [“schizophrenia”] AND [“symptom” OR “cognition” OR “cognitive function”] AND [“MRI”]. Abstracts and main texts were assessed with the following inclusion/exclusion criteria. The inclusion criteria were the following: 1) publications between January 2000 and January 2017, 2) diagnosis of any psychiatric disorders or risk gene, 3) brain structure with volume, concentration, thickness, and surface area, 4) brain function including resting state or task, and 5) including modalities of gene, imaging, and behavior simultaneously. Exclusion criteria were the following: 1) publications including letters, short reports, and brief communication; 2) MRI scanning sequences other than T1, T2, or BOLD; 3) in functional studies, the association between genes, images, and behavior were not directly assessed; and 4) in structural studies, symptoms, cognition, or behavior was not evaluated and collected at the same time window as images were acquired. After excluding 7 studies, 24 studies remained in **Table 1** for functional studies. **Table 1** lists the selected functional papers, and we highlight findings below by symptom/cognitive domains and possible intermediate imaging phenotype. However, most of these imaging genetic studies were done in healthy risk allele carriers.

**Table 1:** Clinical/cognitive domain specific imaging genetic evidence and potential intermediate functional imaging phenotypes.

Clinical/cognitive domains	Risk SNPs/allele	Study	Population	Scan modality	Scanner type	Intermediate imaging phenotype	Specific cognitive task	Risk allele associated functional imaging phenotypes
Working Memory	ZNF804A rs1344706 (A)	Esslinger et al. (52)	HC (115)	BOLD fMRI	Siemens 3T	R DLPFC functional connectivity	N-back task	Increased DLPFC coupling with L hippocampus but decreased coupling within DLPFCs
		Esslinger et al. (53)	HC (111)	BOLD fMRI	Siemens 3T	R DLPFC functional connectivity	N-back task	Increased DLPFC coupling with bilateral hippocampus but decreased coupling within DLPFCs in n-back task
		Linden et al. (54)	HC (43)	BOLD fMRI	Philips 1.5T	Rostral R DLPFC activation	Memory task with Ekman face images	Decreased activation
		Paulus et al. (55)	HC (94)	BOLD fMRI	Siemens 3T	DLPFC functional connectivity	–	Increased DLPFC coupling with hippocampus formation
		Rasetti and Weinberger (56)	SZ (78), US (171) and HC (153)	BOLD fMRI	GE 3T	DLPFC functional connectivity	N-back task	Risk allele carriers’ DLPFC “inefficiency” in the SZ and US group greater than HC
	CACNA1C rs1006737 (A)	Bigos et al. (57)	HC (131/316 in respective tasks)	BOLD fMRI	GE 3T	PFC activation	Emotional face task and n-back task	Increased regional activation
		Paulus et al. (58)	HC (94)	BOLD fMRI	Siemens 3T	DLPFC activation and functional connectivity	N-back task	Decreased task related activation and increased coupling between DLPFC and hippocampus
	ANK3 rs9804190 (C)	Roussos et al. (59)	HC (52)	BOLD fMRI	GE 1.5T	L IFG, L MFG activation	N-back task	Increased regional activation in L IFG and L MFG
	COMT Val158Met	Tan et al. (60)	HC (46)	BOLD fMRI	GE 3T	DLPFC to striatal effective connectivity	Event-related working memory task	Increased DLPFC parietal ‘excitatory’ effective connectivity in Met-carriers
	RGS4 rs951436 (A)	Buckholtz et al. (61)	HC (94)	BOLD fMRI	Siemens 1.5T	R VLPFC connectivity	N-back task	Decreased right VLPFC connectivity to DLPFC and parietal cortex
	COMT X GRM3 epistasis	Tan et al. (62)	HC (29)	BOLD fMRI	GE 3T	DLPFC, VLPFC activation and functional connectivity	N-back task	Inefficient PFC engagement and altered PFC-parietal coupling with COMT Val/Val and GRM3 AA/G
	NRGN rs12807809 (T)	Rose et al. (63)	HC (52)	BOLD fMRI	Philips 3T	Activation in frontal lobe	Block design spatial working memory task	A load-independent decrease in left superior frontal gyrus during task
Episodic memory	CACNA1C rs1006737 (A)	Erk et al. (64)	HC (50)	BOLD fMRI	Siemens 3T	Activation in hippocampus and functional connectivity	3 consecutive memory tasks including coding, recall and face-profession pairs	Decreased activation in hippocampus and various brain regions, and decreased bilateral hippocampus connectivity
		Krug et al. (65)	HC (205)	BOLD fMRI	Siemens 3T	Hippocampus activation	Memory encoding and retrieval task	Decreased activation in hippocampus during task
		Erk et al. (66)	US (188)	BOLD fMRI	Siemens 3T	Activation in hippocampus, DLPFC and functional connectivity	Memory encoding and retrieval task	Replication to previous and decreased activation in DLPFC associated with genetic risk score
	NRGN rs12807809 (T)	Krug et al. (67)	HC (94)	BOLD fMRI	Siemens 3T	Activation in various regions	Memory encoding and retrieval task	Increased activation in L lingual gyrus, ACC and Inhibited deactivation in L precentral gyrus, and L insula during task
Cognitive control/attention	ZNF804A rs1344706 (A)	Thurin et al. (68)	HC (208)	BOLD fMRI	GE 3T	DLPFC and ACC activation and effective connectivity	Modified Flanker task	Decreased PPI connection between DLPFC and ACC
	CACNA1C rs1006737 (A)	Thimm et al. (69)	HC (80)	BOLD fMRI	Siemens 3T	Activation in parietal and frontal lobes	Attention network test including: alerting, orienting and executive control	Decreased activation in R inferior parietal lobule and MFG
	NOS1 rs3782206 (T)	Zhang et al. (70)	HC (ᑔ78)	BOLD fMRI	Siemens 3T	Activation in R IFG and coupling of DLPFC	N-back task and stroop task	Decreased activation in R IFG and reduced connectivity between IFG and DLPFC
Emotion	ZNF804A rs1344706 (A)	Esslinger et al. (52)	HC (115)	BOLD fMRI	Siemens 3T	Functional connectivity of R amygdala	N-back task	Increased functional connectivity between R amygdala and numerous brain regions
	CACNA1C rs1006737 (A)	Bigos et al. (57)	HC (116/131 in respective tasks)	BOLD fMRI	GE 3T	Activation in hippocampus	Emotional memory task, emotional face task	Increased activation in bilateral hippocampus during emotion memory task
	COMT Val158Met	Drabant et al. (71)	HC (101)	BOLD fMRI	GE 3T	Activation in hippocampus and VLPFC	Corticolimbic reactivity task	Increased hippocampus and VLPFC activation and in met/met there was increased limbic and prefrontal regions coupling during emotional face task
	DRD2 rs1076560 (G)	Blasi et al. (72)	HC (24)	BOLD fMRI	GE 3T	Activation and functional connectivity of amygdala and DLPFC	Facial expression task	Increased activation in both regions, and coupling of both of them associated with emotion control scores
	MIR137 rs1625579(T)	Mothersill et al. (73)	HC (98)	BOLD fMRI	Philips 3T	Fronto-amygdala functional connectivity	Face processing task	Increased amygdala connectivity with various regions in frontal lobe
Theory of mind	ZNF804A rs1344706 (A)	Walter et al. (74)	HC (109)	BOLD fMRI	Siemens 3T	Functional connectivity of DLPFC and activation	A theory of mind task judging picture to picture changes	Decreased activation in various brain regions and increased functional connectivity between DLPFC and R precentral gyrus, medial temporal gyrus and L lingual gyrus
		Mohnke et al. (75)	HC (188)	BOLD fMRI	Siemens 3T	Functional connectivity of left temporal parietal junction	Theory of mind task same as above	Increased functional connectivity between left temporal parietal junction and various brain regions

The phenotypic changes in last column corresponds to the risk allele. ACC, anterior cingulate cortex; BOLD fMRI, blood-oxygen-level dependent functional magnetic resonance imaging; DLPFC, dorsolateral prefrontal cortex; HC, healthy controls; IFG, inferior frontal gyrus; L, left; MFG, medial frontal gyrus; PFC, prefrontal cortex; R, right; SZ, schizophrenia; US, unaffected siblings; VLPFC, ventrolateral prefrontal cortex.

**Table 2:** Potential structural imaging genetic phenotypes and possible function association.

Genetic factors	SNPs and risk allele	Study	Population	Scan modality	Scanner type	Imaging phenotype associated with risk allele	Function/symptom implication
ANK3	rs1938526 and rs10994336	Cassidy et al. (76)	First-episode psychosis patients (82)	T1	Siemens 1.5T	Widespread cortical thinning	General cognitive impairment
APOE	e4	Hata et al. (77)	SZ (21)	T1	GE 1.5T	Trend of reduce R hippocampal volume	Memory and cognitive function
BDNF	Val66Met(Met)	Ho et al. (78)	HC (80) and SZ (183)	T1, proton density and T2	GE 1.5T	Reduced hippocampal, temporal and occipital grey matter	Hallucinations. Impaired cognitive functions including working memory, episodic memory and etc.
		Pezawas et al. (79)	HC (214)	T1	GE 1.5T	Reduced hippocampal and prefrontal grey matter volume	Memory, learning, executive function and attention
		Bueller et al. (80)	HC (36)	T1	GE 1.5T	Reduced hippocampal grey matter volume	Emotional reactivity traits and episodic memory
		Aas et al. (81)	Schizophrenia spectrum disorders (48), BD (58), and MDD (3)	T1	Siemens 1.5T	Reduced hippocampal volume	Impaired cognitive functions including working memory and episodic memory
		Carballedo et al. (82)	MDD (62) and HC (71)	T1	Philips 3T	Reduced hippocampal volumes	Met carriers were in line with MDD patients (smaller hippocampal volume)
		Gatt et al. (83)	HC (89)	T1	Siemens 1.5T	Reduced hippocampal and prefrontal volumes	Impaired working memory, depression and anxiety traits
		Gerritsen et al. (84)	HC (275 for 1.5T and 293 for 3T)	T1	Siemens 1.5T and 3T	Reduced anterior cingulate volume	Sensitive to childhood adversity
		Nemoto et al. (85)	HC (109)	T1	Siemens 1.5T	Reduced DLPFC volume	DLPFC reduction related to age and gender
CACNA1C	rs1006737(A)	Wang et al. (86)	HC (55)	T1 and BOLD fMRI	Siemens 3T	Greater gray matter volume in cortico-limbic fronto-temporal region	Decrease functional connectivities from altered structural regions observed during emotion tasks
		Cerasa et al. (87)	HC (57)	T1	GE 1.5T	Increased hippocampal volumes	Executive cognition
COMT	Val158	Honea et al. (88)	HC (151)	T1	GE 1.5T	Reduced hippocampal and DLPFC gray matter volume	Nonlinear dependence of prefrontal neurons on extracellular dopamine
		Mechelli et al. (89)	HC (50)	T1 and BOLD fMRI	GE 3T	Reduced hippocampal volume and decreased activation of parahippocampal gyrus during facial expressions	Emotional processing
		Taylor et al. (90)	HC (31)	T1	GE 1.5T	Reduced temporal lobe and hippocampal volumes	Memory and emotional processing
		McIntosh et al. (91)	SZ (11), High risk subjects (67) and HC (15)	T1 and BOLD fMRI	Siemens 1T	Reduced ACC grey matter volume and increased activation in L PFC and PCC	Increasing sentence difficulty
		Ohnishi et al. (92)	SZ (47) and HC (76)	T1	Siemens 1.5T	Reduced L ACC and R MTG grey matter volume	Mental efforts, working memory, etc.
		Ho et al. (93)	SZ (159) and HC (84)	T1	GE 1.5T and PET	Negative in MRI, but higher frontal lobe activation in performing the one-back task	Working memory and executive function
DISC1	Ser704Cys (Cys)	Gruber et al. (94)	SZ (30) and non-affected family members (52)	T1	Siemens 1.5T	Reduced hippocampal volume	Grey matter reduction shared in family structure
NRG1	HAP_ICE_	Tosato et al. (뽌^95^)	SZ (27)	T1	Siemens 1.5T	Reduced superior temporal gyrus volume	Implications of the language disturbances
		Addington et al. (96)	Childhood onset SZ (78) and HC (165)	T1	GE 1.5T	Risk allele carriers have greater total grey matter and white matter volume in childhood and a steeper rate of subsequent decline in volume into adolescence.	Genetic effects in various cognitive and social function development
ZNF804A	rs1344706 (A)	Lencz et al. (97)	HC (39)	T1	GE 1.5T	Larger total white matter volumes and reduced grey matter volumes in angular gyrus, parahippocampal gyrus, posterior cingulate, and medial orbitofrontal gyrus	Risk allele carrier showed worse visuomotor performance task
		Donohoe et al. (98)	SZ (70) and HC (38)	T1	Siemens 1.5T	Larger hippocampal volumes in patients Larger white matter volume in total, frontal and parietal lobe. Reduced L superior temporal gyrus volume with higher PRS for SZ	No genetic effects were found in the measures of positive, negative or general symptom severity
		Wassink et al. (99)	Schizophrenia spectrum disorders (306) and HC (198)	T1	GE 1.5T		Risk allele carriers also showed severer psychotic symptoms including hallucination and delusion
Polygenic Risk		Ohi et al. (100)	SZ (160) and HC (378)	T1	GE 1.5T		Contributing SNPs located in genes involved in developmental delay and cognitive impairment
		Terwisscha et al. (ᑔ^101^)	SZ (뽌152) and HC (ᑔ142)	T1	Philips 1.5T	Reduced whole brain white matter volume with higher PRS for SZ	SNPs located in neuronal functions are associated with white matter reduction
		Harrisberger et al. (102)	At-risk mental state (43) and first episode psychosis (36)	T1	Siemens 3T	Reduced hippocampal volumes with higher PRS for SZ	First episode psychosis patients have higher genetic risk than the at-risk mental state participants

The phenotypic changes in last column corresponds to the risk allele. ACC, anterior cingulate cortex; BOLD fMRI, blood-oxygen-level-dependent functional magnetic resonance imaging; DLPFC, dorsolateral prefrontal cortex; HC, healthy controls; L, left; MFG, medial frontal gyrus; MTG, middle temporal gyrus; PET, positron emission tomography; PFC, prefrontal cortex; PRS, polygenic risk score; R, right; SNP, single nucleotide polymorphism; SZ, schizophrenia.

Working memory deficit is fundamental and critical in SZ. The most well-studied possible intermediate imaging phenotype was the connection abnormalities between dorsolateral prefrontal cortex and hippocampus (DLPFC-HC). ZNF804A (52, 55, 56) and CACNA1C (58) were associated with DLPFC-HC connection alteration. In the healthy controls, risk allele of ZNF804A was associated with the increased DLPFC-HC connection. COMT (60), regulator of G protein signaling 4 (RGS4) (61), and COMT X glutamate metabotropic receptor 3 (GRM3) epistasis were connected to prefrontal cortex-parietal coupling.

Episodic memory or long-term memory was also often disturbed in SZ. Possible intermediate imaging phenotypes included decreased coupling of the hippocampus–parietal cortex, hippocampus and ventrolateral prefrontal cortex (VLPFC), and bilateral hippocampus. However, the genetic association within this thread is elusive (46). In healthy controls, CACNA1C risk allele carriers showed the decreased activation during recall in decreased coupling between bilateral hippocampus (64). The NRGN rs12807809 was found with increased activation in the left lingual gyrus and decreased deactivation in the left precentral gyrus, cingulate, and left insula during the different stages of memory retrieval (67).

SZ patients often show attention or cognitive control deficits. Disturbances in PFC and DLPFC and connection alterations were the most important issue regarding this deficit. NOS1 risk allele carriers showed reduced inferior frontal gyrus and DLPFC connection associated with attention performance (103). For other risk genes, CACNA1C risk allele carriers showed decreased activation in the right inferior parietal lobule and medial frontal gyrus during an attention task (69). Again, ZNF804A showed association with the anterior cingulate cortex (ACC) and DLPFC coupling during attention and cognitive control (68). During emotional memory, SZ CACNA1C risk allele carriers showed increased activation in the bilateral hippocampus, which was in line with finding in BD (57).

Emotion processing is another important disruption common in SZ. ZNF804A (52) and DRD2 (72) have shown correlation with the amygdala and ACC/medial prefrontal cortex (mPFC) within emotion processing. Increased connectivity between amygdala and VLPFC, which was considered as another intermediate imaging phenotype for emotion processing, has been found in healthy risk allele carriers of the COMT (71) and MIR137 (73).

As part of social cognition that is often impaired in SZ, the theory of mind capabilities tends to help people understand mental states of themselves and others. ZNF804A risk alleles correlated with the PFC and various cortical regions in social information processes (74). Decreased activation in bilateral dorsal medial PFC, the left temporoparietal cortex, left inferior parietal cortex, posterior cingulate, and the left lateral PFC was found while investigating ZNF804A (74). There was also a trend for increased functional connectivity of the left temporal parietal junction with several regions (75).

Rather than a localized abnormality, most findings are notably in line with a “disconnection disorder” (104). Additionally, as noted above, these genes are not specific to risk for schizophrenia but show risk as well for other psychiatric disorders; the common functional impairments showing the genetic relationship in SZ and BD tend to be closely associated with connection disturbances and involve multiple brain regions (46, 105, 106).

### Structural Brain Imaging Genetics Findings in SZ

It is more difficult for researchers to relate risk gene factors, brain structural alteration, and symptoms or cognitive impairments; large numbers of these structural brain imaging genetic studies have conflicting results (10). We reviewed structural brain imaging studies with the relatively clear and consistent symptom or cognitive implications following the criteria we described above. Note that only research involving genetics, structural brain, symptoms, or cognitions and the analysis between them were included. After excluding 8 studies, 27 structural studies remained (see **Table 2**).

Some genes like BDNF are engaged in many cognitive domains that are commonly impaired in SZ, although their associations with SZ *per se* may not be strong. BDNF is essential in nervous system development and prevention of cell loss in various brain regions including the hippocampus, striatum, and more. The Val66Met has been found to be related with reduced hippocampal (107), temporal (78), and frontal volume (80), which may affect various cognitive functions including working memory, episodic memory, executive function, and hallucinations. Its interaction with early life abuse may also result in reduced hippocampal volume in SZ, BD, and MDD (81, 82). As the disease progresses, BDNF is found to be connected with reduced frontal volume and impaired executive function (85, 108).

Other genes may have a closer relationship with SZ, but their imaging genetic findings with brain regions and clinical phenotypes are less consistent. COMT may be involved not only in reduced hippocampal volume but also in reduced cingulate and DLPFC volume, which may potentially affect memory, attention, and executive function (87, 91). Risk allele carriers with rs1006737(A) in CACNA1C show greater gray matter volume in a cortico-limbic and fronto-temporal region but generally in BD (86). The neuregulin 1 gene (NRG1) and its risk haplotype may also contribute to the hippocampal and temporal volume (95, 109). Other critical genes including ANK3 (76), Apoe (77), DISC 1 (110), and ZNF804A (97–99) and more have been found connected to reduced brain volume in hippocampus, cingulate, frontal, temporal, and various brain region volume, suggesting their role in SZ-related cognitive impairment and symptoms.

Polygenic risk score studies also provide imaging genetic evidence for SZ imaging genetic. Temporal volume (100), whole brain white matter volume (101), and hippocampal volume abnormality (102) have been suggested through these approaches.

Overall, the genetic influence on brain structure are widely spread, and their functional or clinical implications are complex. At the moment, the gene to the brain and behavior/symptom links are extensive, affecting many cognitive domains when tested in nonaffected individuals. The specificity of genetic effects on SZ need to be carefully examined, and uncovering better methods to form a link from imaging genetics to clinical phenotypes is important to contribute to the diagnostic issue.

## Limitations and Future Directions

Imaging genetics has contributed greatly to our understanding of the biological mechanism behind psychiatric disorders by revealing the potential association between genetics and imaging phenotypes. The merging boundaries between disorders and subtypes within SZ revealed by imaging genetics will continue to shape the future diagnostic approach of psychiatric disorders. However, it also has inevitable limitations, and the pathways linking genetics, neuroimaging intermediate phenotypes, and clinically assessable phenotypes remain far from clear. A diagnostic system built on imaging genetics requires further research efforts.

### Limitations

Typically, the effect size of candidate gene analyses is rather small and explains limited brain structural or functional variations (111). Alternatively, large sample imaging genetics research often report encouraging findings supporting vast common variations influence on the human brain (112).

However, these findings and even the logic behind imaging genetics has been questioned. Franke et al. mega-analyzed the largest GWAS data for SZ to date from PGC (33,636 cases and 43,008 controls) and eight structural MRI brain measures from ENIGMA (11,840 individuals) to evaluate the relationship between the common variations and SZ-associated subcortical brain regions (113). For instance, the hippocampal volume deficit was thought fundamental in SZ (114). The hippocampus deficits in SZ are one of the most reliable findings of volumetric deficits (44). The ENIGMA analysis identified common genetic variations related to hippocampal volume without regard to disorder (112); the PGC identified common genetic variations highlighted by 108 loci from GWAS, which were thought to play important roles in the etiology of SZ without regard to hippocampal volume (16). Franke et al. did several analyses to investigate the correlation between these genetic and imaging findings. They used linkage disequilibrium score regression to estimate the SNP-based heritability of volumetric measures, computed and compared genetic predisposition scores to volumes, and quantified rank–rank hypergeometric overlap test and listed genetic variants influencing the brain volume. Unfortunately, all these analyses reported no significant results. They also analyzed the 128 index SNPs from PGC and their association with brain volume including the hippocampus, meta-analyses, conjunction analysis and compare the genetic effect sizes for SZ and volumes. Again, these analyses resulted in nonsignificant findings.

Although Franke et al. emphasized that there were several limitations that may result in this null finding, it strongly reminded us to think carefully about the logic of imaging genetics. Brain measures or structural brain deficits believed to be important pathological alterations of SZ may not be induced by those primary genetic causes of SZ as a diagnostic category. They may be reflecting prenatal and later development environmental effects that correlate with but are not specific to SZ, or the diagnostic category of SZ may not be uniformly organized so the large-scale studies of disease risk may have introduced too many heterogeneities.

Instead, the field must consider whether brain volume is a good bridge to look into the genetic influence on disorders. The idea of “intermediate phenotype” succeeded the idea of endophenotype, which was first used by Gottesman and Shields (115). Either structural or functional imaging was believed to be good intermediate phenotypes, as they provide a large amount of data that can show the effect of genes. Although many imaging genetics studies used the concept of intermediate phenotypes to conduct the hypothesis and research flow, they did not fully meet the criteria of intermediate phenotype. To fulfill the criteria, the phenotype must have the following: good psychometric properties, disorder and symptoms related in general population, stable over time, increased expression in unaffected relatives, cosegregation in families, and common genetic influences shown in the disorder. We have to verify whether a chosen brain measure meets each of these criteria.

Hippocampal volumes, in particular, did seem to fulfill these criteria, in that the volumes were more similar in unaffected siblings (116, 117), seemed to decrease with younger disease onset (118), and the smaller volumes were a strong effect in comparing SZ and controls (119). The other brain regions especially caudate and putamen, which showed a small effect size in ENIGMA, would also need to pass these criteria if they are to be used as intermediate phenotypes. However, these brain volume alterations may not be specific to SZ. As for hippocampal volume among psychiatric disorders, it is also affected in MDD (120, 121), obsessive–compulsive disorder (122), and attention deficit hyperactivity disorder (123). Other than psychiatric disorders, cardiovascular disease, diabetes, hypertension, obesity, physical activity, and various somatic factors may also play a role in modifying hippocampal volume to different extents (124, 125). The hippocampus is vulnerable to various environmental factors from the prenatal stage throughout the lifetime, which makes the hippocampal structure sensitive to neurodisruption but not necessarily specific to SZ (126). The specificity of these altered brain volume will need careful examination before being considered as part of SZ’s pathology in complicated clinical situations.

Another approach is to reconsider other imaging intermediate phenotypes bridging genetics and SZ. For example, there are various anatomic measures other than volumes that should be assessed for genetic effects (127, 128). Gray/white matter density, cortical thickness, cortical folding, cortical surface area, and white matter integrity are potential useful intermediate phenotypes from which to choose. However, although it may also be difficult to fully grasp, the functional implication of these brain measures and their compatibility with genetics will need further investigation (129). As for these other brain volumes and functional measurements, their stability, situation in unaffected relatives, families, and general population will need to be further investigated to answer the criteria question as well as their specificity to the diagnosis or clinical subgrouping.

It may be helpful to expand the genetic modality of imaging genetic study. More heritability could be captured by involving rare variance and chromosome structural variations like CNVs (38). Both options will need better imaging genetic analysis methods and models.

### Future Directions

The current review summarized genetics, imaging, and imaging genetics in schizophrenia to date. Imaging genetics may continue to shape the future conceptualization of SZ and psychotic disorders in both clinical and research field.

One future direction is collecting a large number of genetic effects. The method applied by Arnedo et al. is promising in coupling both genetic and phenotypic clusters, but it may need to establish its association with imaging data or physiological measures. The clustering method shows great complexity, while its compatibility with neuroimaging is unknown. The other polygenic method like polygenic risk score is also promising. However, it will call for more common variations and the combination with other data (e.g., the B-SNIP biotype study).

Parallel independent component analysis (pICA) may be another useful tool in this field. This method allows independent components from two modalities to be identified simultaneously, and the association between these two modalities is optimized. pICA is designed to be totally theoretically blind and data-driven, but pICA with reference allows *a priori* knowledge as the reference to improve robustness. For instance, a set of genes from the same pathway can be used as a reference to highlight their effect on certain brain components as well as behavioral data (130). Chen et al. used pICA and reported that the gray matter density of frontal, precuneus, and cingulate regions might potentially be affected by various genes participating in synaptic plasticity, axon guidance, and molecular signal transduction (131).

Another possible direction is refining the clinical assessment tools to better complement imaging genetics. As raised in the B-SNIP study, a series of symptom rating scales including the Global Assessment of Functioning scale, the Positive and Negative Syndrome Scale, the Young Mania Rating Scale, the Montgomery–Åsberg Depression Rating Scale, the Schizo-Bipolar Scale, and the Birchwood Social Functioning Scale were obtained from the participants. These measures were not able to distinguish SZ, BD, and SAD significantly or contribute much in the building of biotypes (132). Imaging genetic compatible comprehensive symptom scales are needed. These scales are not aiming at distinguishing traditional diagnostic groups or a certain diagnostic group usage. However, they would provide comprehensive clinical profiles “scanning” the symptom domains (2). Some scales like the Symptom Checklist-90 (SCL-90) and its revised version (133) might be worth trying (134). More detailed multidimensional symptoms reflecting scale need to be developed to fit the need of imaging genetics and clarify the path linking genotypic variation, intermediate brain imaging, and clinical phenotypes.

Finally, future research will need to be enhanced by improving power and replicability. Studies with small number of subjects (below 100 participants) will be able to show moderate power with effect size of 0.5. However, it is critical to replicate them independently with same genetic variants, imaging, and behavioral measurements, and direction of the effects by Carter et al. (135). It is also argued, in such studies, null results or conflicting associations with failed replication should still be considered for publications as potentially informative or innovative studies (6, 136). In this case, meta-analytic studies addressing the conflicted results and the issues of publication bias will help to avoid the misleading information potentially generated from small sample research results (6, 137).

## Author Contributions

All authors listed have made substantial, direct, and intellectual contribution to the work and approved it for publication.

## Funding

WJ and JT were supported in this work by a grant from the National Institute of Mental Health (R01 MH094524).

## Conflict of Interest Statement

The authors declare that the research was conducted in the absence of any commercial or financial relationships that could be construed as a potential conflict of interest.
